# “Mine did not breastfeed”, mothers’ experiences in breastfeeding children aged 0 to 24 months with oral clefts in Uganda

**DOI:** 10.1186/s12884-021-03581-3

**Published:** 2021-01-30

**Authors:** Maureen Nabatanzi, Gloria Kimuli Seruwagi, Florence Basiimwa Tushemerirwe, Lynn Atuyambe, David Lubogo

**Affiliations:** Makerere University College of Health Sciences, School of Public Health, New Mulago Hospital Complex, P.O. Box 22864, Kampala, Uganda

**Keywords:** Breastfeeding, Experiences, Children, Cleft lip, Cleft palate, Support

## Abstract

**Background:**

Appropriate breastfeeding is vital for infant and young child nutrition. Annually, oral clefts affect 0.73 per 1000 children in Uganda. Despite this low incidence, children with a cleft face breastfeeding difficulty which affect their nutrition status. In addition, knowledge on maternal experiences with breastfeeding and support is limited. We explored maternal perceptions, experiences with breastfeeding and support received for their children 0 to 24 months with a cleft attending Comprehensive Rehabilitative Services of Uganda (CoRSU) Hospital.

**Methods:**

This cross-sectional study combined quantitative and qualitative methods. We consecutively recruited 32 mothers of children with a cleft aged 0 to 24 months attending CoRSU hospital between April and May 2018. A structured questionnaire collected data on breastfeeding practices and device use (*n* = 32). To gain a broad understanding of mothers’ perceptions and experiences with breastfeeding and support received, we conducted two Focus Group Discussions (in each, *n* = 5), and 15 In Depth Interviews. Descriptive statistics were analyzed using SPSS software. Qualitative data were analyzed thematically.

**Results:**

Of the 32 children with a cleft, 23(72%) had ever breastfed, 14(44%) were currently breastfeeding, and among those under 6 months, 7(35%) exclusively breastfed. Of 25 mothers interviewed in IDIs and FGDs, 17(68%; IDIs = 8/15, FGD1 = 5/5 and FGD2 = 4/5) reported the child’s failure to latch and suckle as barriers to breastfeeding. All ten mothers who used the soft squeezable bottle reported improved feeding. Nineteen (76%) mothers experienced anxiety and 14(56%), social stigma. Family members, communities and hospitals supported mothers with feeding guidance, money, child’s feeds and psycho-social counselling. Appropriate feeding and psycho-social support were only available at a specialized hospital which delayed access.

**Conclusions:**

Breastfeeding practices were sub-optimal. Mothers experienced breastfeeding difficulties, anxiety and social stigma. Although delayed, feeding, social and psycho-social support helped mothers cope. Routine health care for mothers and their children with a cleft should include timely support.

**Supplementary Information:**

The online version contains supplementary material available at 10.1186/s12884-021-03581-3.

## Background

Oral clefts or “orofacial clefts” (Cleft lip and or Cleft palate) are congenital deformities that affect the lips, oral cavity and or nostril as a result of poor fusion of tissues during early pregnancy [1]. Non-syndromic clefts occur in isolation while syndromic clefts are part of another congenital anomaly or medical condition, for example, Pierre Robin sequence, and Constricting ring syndrome [2].

Globally, clefts affect 0.9 newborns per 1000 live births; the prevalence varies geographically from as high as 2 per 1000 in Japan, to as low as 0.2 per 1000 in South Africa [3]. Among African populations, Kenya reports 1.65 per 1000 while Uganda reports 0.73 per 1000 [4, 5]. In 2016, 163 children with a cleft reported to Comprehensive Rehabilitative Services in Uganda Hospital (CoRSU); 90% percent of them were below 12 months of age [6].

Despite their low prevalence, the occurrence of clefts in children is associated with malnutrition due to feeding difficulties. Being malnourished compromises the child’s growth, development and weight gain needs which are critical for successful cleft repair surgery [7, 8]. Without cleft repair surgery, feeding difficulties in these infants include insufficient suction during breastfeeding, nasal regurgitation, and reduced food intake [7]. In addition, infant feeding can be affected by mothers’ negative perceptions, poor societal support, stigma and lack of guidance from health care professionals [9].

Care and management of clefts generally aims to improve feeding ability, surgical repair and psycho-social support. Feeding interventions include: early guidance on breastfeeding positioning and breast milk expression, provision of feeding devices such as: soft squeezable bottles, Nasal Gastric (NG) tubes or nifty cups, and weight monitoring [7, 10]. The timing and choice of procedure for surgical repair depends on the country, the plastic surgeon, the clinical presentation, age and nutritional status of the child. In the United States and Uganda, cleft lip repair is done between 2 and 3 months old and 5 Kilograms weight while the cleft palate repair is done between 6 and 12 months old [6, 11]. In Uganda, surgical repair is sometimes delayed because the child is malnourished [8]. The World Health Organization (WHO) recommends that parents to children with a cleft receive emotional support and advice to cope with the risk of psychological distress and societal stigmatization of the disability [12].

The WHO and Uganda’s infant and young child feeding (IYCF) guidelines recommend the following practices: early initiation of breastfeeding where children receive breast milk within one hour of birth, exclusive breastfeeding from 0 to 5 months, and continued breastfeeding from 6 to 24 months or beyond in addition to appropriate and adequate complementary foods [13]. In the general Ugandan population, 66% of children 0 to 5 months are exclusively breastfed, 82% breastfeed from 12 to 17 months and 50% breastfeed from 18 to 23 months [14].

Low breastfeeding rates have been reported among children with a cleft. In Denmark, only 22% of children with a cleft were exclusively breastfed for at least 4 months [15]. In a 2017 Ugandan study, 50% of children with a cleft aged less than 4 months assessed were breastfeeding [16]. This poor feeding practice contributed to the high prevalence of wasting of 74%, which was higher than the 2016 national overall of 8% among children under 6 months of age [14, 16].

Uganda has no published guidelines to address the special healthcare needs of children with a cleft. The national IYCF policy by the Ministry of Health (MoH) has gaps in feeding interventions for children with cleft [17]. In addition, there are barriers to accessing feeding, psycho-social and health services. Children with a cleft from Uganda, and sometimes from the neighboring countries of South Sudan and the Democratic Republic of Congo primarily rely on referral to one national referral hospital and two specialist hospitals. One of the specialist hospitals providing feeding, psycho-social and cleft repair surgical services in Uganda is CoRSU hospital [6].

Furthermore, published literature on breastfeeding experiences and support among children with a cleft in Uganda is limited [14, 16]. In this study, we describe mothers’ perceptions, experiences with breastfeeding and support given to children with cleft in CoRSU a private specialized hospital in Uganda.

## Methods

### Study design

This cross-sectional design integrated quantitative and qualitative methods; this allowed for description of breastfeeding practices, and an in-depth exploration of maternal perceptions, experiences, and support. In a quantitative survey, we collected data on child’s history of: breastfeeding, pre-lacteal and breast milk alternative use, and feeding device use. The sample size was achieved using the census method which lists all available elements in a population and it is applicable for rare groups like this [18]. We used a consecutive sampling strategy, where each person who meets the inclusion criteria is selected until the sample size is achieved. Subsequently, each consecutive mother-child pair with a cleft who attended CoRSU hospital during a two-month survey period was approached for enrolment. The survey sample amounted to 32 mother-child pairs with a cleft (*n* = 32). The researchers believed this consecutive sample was more representative of the target population than one from convenience sampling. During the same time period, we conducted Focus group discussions (FGD) and In depth interviews (IDI) to explore perceptions about breastfeeding and support received. This triangulation of methods enabled us to check out the consistency of findings generated. We purposively selected mothers to children with a cleft to allow for deep exploration of their lived experiences with breastfeeding. Out of the 32 mothers that were recruited during their visit to CoRSU during the study period, 25 mothers consented to be interviewed in two FGDs, each with 5 mothers, (*n* = 10) and 15 IDIs (*n* = 15).

### Study site

The study was conducted at CoRSU hospital in Uganda located in Wakiso District in Uganda. The hospital was purposefully selected because it provides specialized feeding, psycho-social and cleft repair surgical services for children with a cleft referred from all over Uganda. Feeding support services include: screening for and treatment of malnutrition, and provision of breastfeeding support to the parents. The psycho-social support involves individual and group counselling.

### Study participants

The study included mother-child pairs of children 0 to 24 months old, born with a cleft and presenting at CoRSU hospital, Uganda between April and May 2018. The children were either waiting for cleft surgical repair, recovering from one, or between surgeries. All included mothers were considered for recruitment into FDGs and IDIs because of their common characteristic of having children with a cleft. Mother-child pairs where the mothers were unwilling or unable to participate in the study due to psychological incapacitation or child’s death were excluded. Children with Pierre Robin sequence and Constricting ring syndrome where included because their breastfeeding practice in Uganda has not been previously documented.

### Study tool and variables

All tools were translated into a local language, Luganda by the team and verified by a language specialist (See supplementary files: Feeding practices questionnaire, In Depth Interview guide and Focus Group Discussion guide). All interviews were conducted in English or Luganda according to participant preference. The survey on breastfeeding practices adapted and used a structured questionnaire from the WHO recommended measures of IYCF indicators that was previously used in Kampala [19, 20] (Feeding practices questionnaire). Breastfeeding variables were: ever breastfed; currently breastfeeding- proportion of children 0 to 24 months fed on breast milk; exclusive breastfeeding- proportion of children 0 to 5 months fed on breast milk alone; continued breastfeeding- proportion of children 6 to 24 months fed on breast milk; and duration of breastfeeding- the period for which a child was fed on breast milk. Pre-lacteal feeds were any feeds given to children before initiating breastfeeding while breast milk alternatives were any feeds given to replace breast milk. Feeding devices referred to use of feeding tools such as: spoon, cup, bottle with nipple, NG tube or soft squeezable plastic bottle. Both FGDs and IDIs had interview guides (FGD guide and IDI guide), were audio recorded had a moderator and note taker. In FGDs, we discussed and sought consensus on maternal group experiences with breastfeeding children with a cleft and explored support received [21]. In IDIs, we explored detailed individual maternal perceptions on their breastfeeding and support experiences [21]. Feeding support was any feeding guidance given to mothers such as: counselling on breastfeeding and provision of devices and feeds. Psycho-social support was any emotional reassurance.

### Data management and analysis

All questionnaires were checked daily for errors and the information verified with the participants. Quantitative data were entered and cleaned in EpiData (version 3.1, EpiData Association, Odense, Denmark). Data were then analyzed into percentages using SPSS (version 16.0: SPSS Inc., Chicago, IL). Audio data were transcribed verbatim [22], and translated into English by two team members fluent in both English and Luganda. Thematic content analysis started with thorough reading of transcripts to identify basic features of the data that we organized as explicit codes and defined; these were written in a code book [22]. We used the codes to group text into segments whose meanings were similar to the codes. From these, we identified underlying patterns which were synthesized and grouped into basic themes [22]. Basic themes represented groups of the lowest-order meanings or responses evident in the text [23]. We summarized and grouped together similar basic themes into more abstract principles to form categories called organizing themes [23]. We then merged similar organizing themes into categories of principal or main meanings concerning breastfeeding and support experiences called global themes. Global themes included: breastfeeding difficulties, devices, feeds, negative attitudes, social stigma and support received [23]. Global themes were interpreted by identifying co-occurrence across IDIs and FDGs.

## Results

### Demographic characteristics

The questionnaire was administered to 32 mothers of children with a cleft whose characteristics are shown in Table 1. The average maternal age was 29 (SD = 7) with a range of 17 to 42 years.

**Table 1:** Demographic characteristics of the mother-child pairs with a cleft (*n* = 32)

Child’s age	n (%)	Child’s type of cleft	n (%)
0 to 5 months	20 (63)	Cleft Lip	11 (34)
6 to 11 months	8 (25)	Cleft Palate	3 (9)
12 to 24 months	4 (13)	Cleft Lip and Palate	18 (56)
**Child’s sex**		**Child’s congenital anomalies**	
Male	18 (56)	None	29 (91)
Female	14 (44)	Pierre Robin Sequence	2 (6)
**Birth weight**		Constricting ring syndrome	1 (3)
< 2.5Kg	3 (9)	**Maternal residence**	**n (%)**
≥ 2.5Kg	28 (88)	Eastern Region	9 (28)
Unknown	1 (3)	Western Region	5 (16)
**Term at birth**		Central Region	18 (56)
Before 37 weeks	3 (9)	**Maternal education**	
At or after 37 weeks	29 (91)	None	1 (3)
**Delivery location**		Primary	14 (44)
Health Facility	28 (88)	Secondary	9 (28)
Home	4 (13)	Tertiary	8 (25)

Of the 32 mothers, 25 were recruited to participate in IDIs and FGDs, characteristics of their children are shown in Table 2.

**Table 2:** Characteristics of children with a cleft whose mothers participated in IDIs and FGDs

Qualitative method	Child’s cleft type			Child’s age group		
	Cleft Lip	Cleft Lip and Palate	Cleft Palate (with syndrome)	0–5 months	6–12 months	13–24 months
**In Depth Interviews (*n*** **= 15)**	6	6	3	8	4	3
**Focus Group Discussion 1 (n = 5)**	2	2	1	3	0	2
**Focus Group Discussion 2 (n = 5)**	1	3	1	2	2	1

### Breastfeeding: practices and mothers’ experiences

**Fig. 1:**
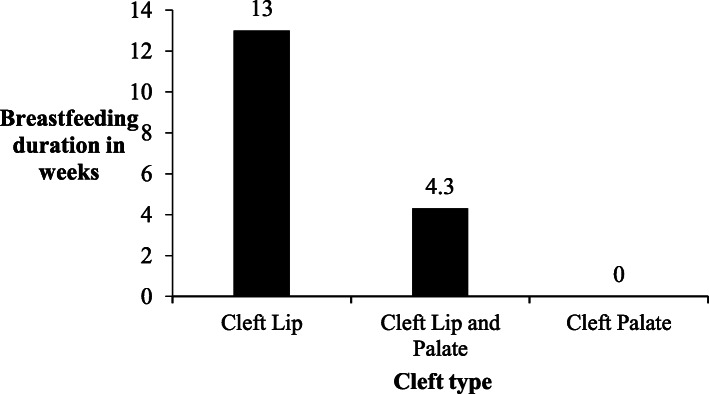
Mean breastfeeding duration according to child’s cleft type

Among 32 children 0 to 24 months with a cleft, 23 (72%) had ever breastfed and 14 (44%) were currently breastfeeding. Among 20 children with a cleft aged 0 to 5 months, 7(35%) breastfed exclusively. None of the 4 children with a cleft aged 12 to 24 months were breastfeeding. The duration of breastfeeding at the time of the study varied according to the child’s cleft type (Fig. 1).

Twenty-five [24] mothers shared their breastfeeding experiences in 15 IDIs (*n* = 15) and 2 FGDs (FGD1 = 5 and FGD2 = 5). Of these, 17 (68%; IDIs = 8/15, FGD1 = 5/5 and FGD2 = 4/5) mothers mentioned not knowing how to breastfeed, the child’s failure to latch or position onto the breast and difficulty suckling as the main feeding difficulties they experienced. Child’s failure to latch onto the breast was also the main reason mothers gave for discontinuing breastfeeding.

In FGD2, four of the five mothers had children with a cleft lip and palate (CLP) or a cleft palate (CP) and struggled to breastfeed; mother #2 whose child had CLP shared that:

“*Mine did not breastfeed. The challenge was that she couldn’t latch onto the breast because the cleft opening was very wide and she could not latch”.*

Breastfeeding was also complicated by difficulties in expressing breast milk and poor breast milk supply as reported by 12 (48%; IDIs = 7/15; FGD1 = 2/5 and FGD2 = 3/5) mothers. A mother to a child with a cleft palate with constricting ring syndrome shared that:

“*I tried expressing breast milk manually using my hands but it was painful. I was advised to buy a breast pump which I did. I tried to maintain my breast milk supply by eating and drinking but it did not work and my breast milk dried up”* FGD1 Mother #1.

General feeding difficulties reported in all FGDs and IDIs were chocking on milk, milk spillages and nasal regurgitation. These caused the children to get frustrated, cry and refuse to continue feeding. Mothers to children with an isolated cleft lip reported less difficulties than those with a cleft lip and palate, and cleft palate. Of the nine mothers whose children had an isolated CL, seven (78%; IDIs = 5/15, FGD1 = 0/5 and FGD2 = 2/5) of them had no breastfeeding difficulties. One mother to a child with a CL shared:

*“Mine breastfeeds. When I had just given birth… the lip couldn’t hold the breast well, so you had to hold for him to feed but now he can hold the breast himself.”* IDI #1:

### Pre-lacteal feeding and breast milk alternatives

As an alternative to breast milk, some mothers resorted to poor alternatives such as sugar, glucose, water and porridge to feed their children with a cleft. Of the 32 children with a cleft assessed quantitatively, 19 (59%), were currently feeding on cow’s milk. Other alternatives to breast milk were porridge (25%), water (16%) and other liquids (6%). Only one child with a cleft (3%) was feeding on infant formula. In all FGDs and IDIs, the general perception was that feeds for the children were expensive and that formula milk was more expensive than cow’s milk.

Mother #4 in FGD 2 said, “*I bought formula milk for about two months, but it was too expensive. So, I started buying and mixing 1 liter of cow’s milk plus soya and rice porridge to feed her, this was before she was 6 months old.”*

### Feeding device usage

Of the 32 mothers to children with a cleft assessed quantitatively, 24 (75%) used at least one device in feeding their children to cope with the feeding difficulties. These included: spoon, cup and ordinary bottle with nipple which are readily accessible from local shops. Soft squeezable bottles, NG tubes and syringes were got from hospitals. Of the 24 mothers that used devices, 16 (66%) preferred the soft squeezable bottle over other devices. All 10 mothers (IDIs = 5/15, FGD1 = 3/5 and FGD2 = 2/5) who received soft squeezable bottles reported improved feeding because it allowed them to regulate the flow of milk to the child’s mouth. However, all five mothers (IDIs = 3/15, FGD1 = 0/5 and FGD2 = 2/5) whose children received NG tubes reported discomfort and pain as described by this mother to a child with CLP:

“*The NG tube we got at the maternity hospital was painful, he was always crying and I also felt restless and dejected. But when I arrived at CoRSU, the staff gave me a soft bottle and it has helped my child to grow. It is soft, you just squeeze it gently for him, and it is so good.”* FGD1, Mother #4

### Feeding support

Perceptions towards support received to help them feed their children with a cleft varied among the 25 mothers. Seven (28%, IDIs = 5/15, FGD1 = 0/5 and FGD2 = 2/5) mothers indicated that before going to CoRSU, other hospitals had not given them adequate feeding guidance. At CoRSU, all 25 mothers reported receiving guidance on breastfeeding positioning, expressing, preparation of alternative feeds using cow’s milk, provision of NG tubes or soft squeezable bottles and treatment for malnutrition. In IDI #2, a mother to a child with CLP said,

“*This boy was born heavy with 3.5 kilograms then he lost weight to 1 kilogram because all the hospitals I went to could not help me. By the time my brother directed me to CoRSU, he was 1 kilogram and really small.”*

In addition, in IDI #7, another mother to a child with CLP reported her positive experience at CoRSU:

“W*e were admitted as in patients and taught how to feed the child, I learnt how to express breast milk and how to prepare milk feeds when the breast milk dried out…”*

### Negative attitudes and social stigma

In addition to breastfeeding difficulties, 19 (76%; IDIs = 12/15, FGD 1 = 5/5 and FGD 2 = 3/5) of the 25 mothers interviewed expressed having feelings of anxiety, anger, dejection, trauma, bitterness, self-pity and fear of being rejected by society because of their child’s cleft. They saw their child’s condition as a problem, and some even considered abandoning the child all together. One mother to a child with a CLP described in IDI #12 that:

*“When I gave birth to him, he was scary to look at and as a result I hid him. I felt defeated, like I had nothing to do because after all he was my own child.*”

Half of the mothers (IDIs = 7/15, FGD 1 = 4/5 and FGD 2 = 3/5) experienced stigmatization from their families and communities which affected their ability to cope with their child’s disability. These two mothers (IDI #15 and IDI #14) shared their experiences with stigma from family members:

*“We had disagreed with my husband over the child’s condition, so he had abandoned all care of the child. He never provided any money, or bought feeds, he did not care for us.”*

*“The biggest challenge I have faced is from my husband’s family. From when I delivered this child, they have been hostile towards the child’s condition because it is the first of its kind in our family.”*

One of the three mothers in FGD2 described her experience with stigma from community:

“*Whenever someone would see the child, they would get shocked and exclaim, ‘What is wrong with this child? The child is deformed!’ This would greatly embarrass me among the community members. I started to fear and even now I still cover up my child and I still worry about having to travel with her.”*

### Social support mechanisms

Eighteen (72%; IDIs = 11/15, FGD1 = 3/5 and FGD2 = 4/5) mothers reported receiving support from their families, communities or hospitals. The nature of social support mothers reported included: money, child’s feeds, child’s clothes, emotional and health related support. The mother in IDI #2 shared that:

“*…when I returned home I found my husband had got another woman and could not give any child support. So the church helped me with some money and other people promised to get me milk, sugar and some transport money. Right now, I am living at my parents’ home.*”

However, seven mothers (28%; IDs = 4/15, FGD1 = 2/5 and FGD2 = 1/5) were denied social support by either their families or communities. In FGD1, 4 of 5 (80%) and in FGD2, 2 of 5 (50%) mothers did not receive any support from their husbands. Receiving psycho-social services from the health workers was also emphasized. Nineteen of the 25 (76%) mothers interviewed reported that counselling, and discussions in mother support groups reassured them and helped them cope with the breastfeeding difficulties and negative feelings of having a child with a cleft. However, mothers shared their disappointment about not getting psycho-social support early at the maternity hospitals where they delivered because this delay had put them at risk of anxiety and depression.

## Discussion

Most children in our study had ever breastfed although less than half of those younger than six months were exclusively breastfeeding. The main mother-child barriers to breastfeeding were failure to attach, latch and suckle due to the cleft disability. Mothers received feeding support in form of guidance and devices that helped them improve their children’s feeding. Most mothers experienced personal negative attitudes and social stigma. Social and psycho-social support from family, communities and hospitals helped them cope with their child’s cleft. Mothers to children with a cleft need early breastfeeding, social and psycho-social support to appropriately breastfeed and cope with the disability.

### Breastfeeding and feeding device usage

Most children (72%) in this survey had ever breastfed, less than half were currently breastfeeding and among those under 6 months old, exclusively breastfeeding (EBF). None of the children with a cleft in our study breastfed beyond 12 months. It is not uncommon for mothers to children with a cleft to attempt to breastfeed. Studies on children with a cleft in Brazil, Norway and Uganda reported similar EBF rates of less than half [16, 24, 25]. Breastfeeding practices are better in the general Ugandan population; the 2016 national demographic and health survey reported an EBF rate of 66% while 82% (ages 12–17 months) and 50% (ages 18–23 months) of children were breast fed [14]. However, these national health surveys exclude children with a cleft and the methodology used complicates direct comparison with this study’s cleft group.

Mothers in our study struggled to attach their children onto the breast and the children failed to latch and suckle. They also did not know how to breastfeed a child with a cleft and had difficulty expressing. These difficulties could explain the poor breastfeeding practices. In children with a cleft, difficulty creating negative intraoral pressure which in turn affects attaching to the breast causes the child to fail at maintaining a stable feeding position. These ultimately affect the mother’s let-down reflex and finally milk extraction by the child [10, 26]. Children with a Cleft Lip (CL) had the longest average breastfeeding duration of 13 weeks while children with Cleft Palate (CP) did not breast feed. Cleft type and severity are determinants of breastfeeding outcomes and children with a CP and more so with a complete CLP face additional disadvantages in creating a seal around the oral cavity [10]. The three [3] children with a CP in this study also had associated congenital anomalies (Pierre Robin Sequence and Constricting ring syndrome) which could have further disadvantaged their feeding.

Breastfeeding plays a preventive role in reducing morbidity and mortality from childhood infectious diseases [27]. In children with a cleft, breastfeeding further reduces the risk of infections like otitis media and promotes proper development of oral sensory motor system structures [28, 29]. In the absence of breastfeeding, WHO’s guidelines stipulate that, “commercial infant formula is an option when it is available, affordable, can be safely used, and provides a nutritional advantage over animal milk” [30]. However, in Uganda’s low income setting, not breastfeeding is a disadvantage because breast milk is the only nutritious option for children if their mothers cannot afford appropriate breast milk substitutes like infant formula. In our study, cow’s milk was the main breast milk substitute and was preferred over infant formula because it was the most affordable option for mothers. Mothers reported giving pre-lacteals and inferior breast milk substitutes. These improvised feeds can predispose their children to malnutrition early in life. In a 2012 Ugandan study, 32% of children were fed on pre-lacteals like home-made sugar water which increased their risk of malnutrition; consequently 33% of them were stunted and 13% underweight [31].

To improve feeding, mothers reported using multiple devices and preferred the soft squeezable bottle because they could regulate the flow of milk into the child’s mouth. It is widely recognized that multiple feeding devices including tubes, bottle nipples, cups, spoons, droppers and syringes can improve feeding among children with a cleft [7, 24, 32].

### Feeding support

Most mothers received feeding support at the specialized hospital- CoRSU. The guidance on breastfeeding positioning, expressing, preparation of alternative feeds, provision of NG tubes and soft squeezable bottles, and treatment for malnutrition they received is similar to the recommendation in the United States of America and Brazil [7, 33]. However, in Uganda’s setting, guidance received at maternity hospitals was inappropriate and by the time mothers were referred to CoRSU, their children had lost weight. Mothers to children with a cleft need early and continuous feeding guidance from specialist health workers [7, 33].

### Negative attitudes, stigma and social support

Most mothers experienced anxiety, dejection and self-pity towards their child’s cleft and this was compounded by the social harassment and rejection from their families and communities. When combined with the breastfeeding difficulties, these negative experiences could have further disadvantaged breastfeeding. Negative attitudes among parents, social stigmatization and exclusion because of clefts has been reported in Uganda and globally [34, 35]. In this study, mothers generally received support in the form of: money, help with feeding and caring for the child and emotional comfort from families or communities. Although they could only access it at CoRSU, psycho-social support like counselling and support groups helped mothers cope with anxiety. Previous findings in Uganda identified a gap in psycho-social services for families and communities with children with a cleft and recommended counselling of the affected [34]. Emotional support and advise from family members and professionals can lower stress and help mothers cope [12, 25, 36].

The rich description of mothers’ breastfeeding experiences addresses a knowledge gap in feeding children with a cleft in Uganda. However, the survey had a small sample size (*n* = 32) because of the low prevalence of clefts, the children’s age group (0 to 24 months) and the short study period (April to May). To mitigate this, we used the consecutive sampling method such that all mother-child cleft pairs that visited the hospital during the study period were approached for enrollment. Previous studies on clefts in Uganda had similar sample sizes; Kesande et al., (2014) had *n* = 20 while Tungotyo et al., (2017) had *n* = 44 [16, 34]. In addition, the sample was selected from a specialized hospital such that mother-child cleft pairs who did not access care at the hospital were not considered which may have introduced a selection bias. However, we chose CoRSU because it is the main referral center for cleft care in Uganda. Our findings should not be generalized to all mothers and their children with a cleft in Uganda. Further research on impact of the current breastfeeding guidance on nutrition status of children with a cleft will generate evidence for appropriate feeding interventions applicable in our context.

## Conclusions

The breastfeeding practices in this group of children with a cleft were sub-optimal, contrary to the WHO and national breastfeeding recommendations. Mothers experienced difficulties in attaching their children to the breast as the children could not latch and create suction. Feeding devices and guidance at CoRSU helped mothers to improve feeding. Although mothers experienced anxiety and social stigma, social and psycho-social support from family, communities and hospitals helped them cope with the difficulties.

Uganda’s MoH should review the national feeding guidelines to include breastfeeding guidance and feeding devices for mothers and their children with a cleft. In addition, all hospitals should be equipped to provide psycho-social support to mothers of children with a cleft. Mother support groups can help mothers to share their experiences and suggest coping strategies. These services should be timely, preferably starting at the maternity hospital.

## Supplementary Information

**Additional file 1.** Feeding practices questionnaire. Questionnaire for determining the feeding practices among children with oral clefts at CoRSU Hospital.

**Additional file 2.** In Depth Interview guide. In Depth Interview guide for interviewing mothers (or fathers/family members) on their experiences and challenges in feeding their children with oral clefts.

**Additional file 3.** Focus Group Discussion guide. Focus Group Discussion guide for discussing with mothers to children with oral clefts on feeding assistance, healthcare services and social support received.

## Data Availability

The datasets used and/or analyzed during the current study are available from the corresponding author on reasonable request.

## Abbreviations

EBF: Exclusive Breastfeeding
CL: Cleft Lip
CP: Cleft Palate
CLP: Cleft Lip and Palate
CoRSU: Comprehensive Rehabilitative Services of Uganda Hospital
FGD: Focus Group Discussion
IDI: In depth interview
IYCF: Infant and Young Child Feeding
MoH: Ministry of Health
NG: Nasal Gastric
WHO: World Health Organization
