# Supplementary material for: “Mine did not breastfeed”, mothers’ experiences in breastfeeding children aged 0 to 24 months with oral clefts in Uganda
Source: BMC Pregnancy Childbirth. 2021 Jan 30;21:100. doi: 10.1186/s12884-021-03581-3 (PMC7847043; doi:10.1186/s12884-021-03581-3)
Supplement: Supplementary file 1 — Additional file 1. Feeding practices questionnaire. Questionnaire for determining the feeding practices among children with oral clefts at CoRSU Hospital. [file 12884_2021_3581_MOESM1_ESM.docx]

**Feeding practices questionnaire**

Questionnaire for determining the feeding practices among children with oral clefts at CoRSU Hospital.

**Note:** Complete questionnaire by circling the correct response and recording the code of the response in the last column on the right.

| Date (DD/MM/YY) |  |
| --- | --- |
| Questionnaire code |  |
| Name of interviewer |  |

**Section 1: Maternal and child characteristics**

| **NO.** | **QUESTIONS** | **RESPONSES** |  | **CODE** |
| --- | --- | --- | --- | --- |
| 1 | HOW OLD ARE YOU? (write age in years)  Olina emyaka emeka? | ………………………….. |  |  |
| 2 | WHEN WERE YOU BORN? (dd/mm/yy)  Wazaalibwa ddi? | ……../………../……… |  |  |
| 3 | WHAT IS YOUR CURRENT ADDRESS? (Region in Uganda)  Obeera mu Kitundu kyi ekya Uganda? | Northern  Eastern  Western  Central | 1  2  3  4 |  |
| 4 | HAVE YOU EVER ATTENDED SCHOOL?  Wasoma ko? | Yes  No | 1  2 |  |
| 5 | WHAT IS THE HIGHEST LEVEL OF EDUCATION YOU ATTAINED?  Wasomakutuuka ku ddala kyi? | Primary  Secondary  Tertiary  None  N/A | 1  2  3  4  5 |  |
| 6 | WHAT IS YOUR OCCUPATION?  Okola mulimu kyi? | Civil servant  Paid private employee  Self employed  Not employed  Student | 1  2  3  4 |  |
| 7 | WHEN WAS THIS CHILD BORN? (dd/mm/yy)  Omwana ono yazaalibwa ddi?  Fill in the Child’s age in completed months | ..……/.……./…………  ………………… |  |  |
| 8 | WHAT IS THE SEX OF THIS BABY?  Wa kikula kyi? | Male  Female | 1  2 |  |
| 9 | OF THE CHILDREN YOU PRODUCED, WHAT POSITION IS THIS CHILD? (Probe for number of children born before)  Mu baana bewazaala, ono omwana wakumeka? | ………………... |  |  |
| 10 | WHERE DID YOU DELIVER THIS CHILD FROM?  Omwana ono wamuzalila wa? | Health facility  Home  Traditional birth attendant | 1  2  3 |  |
| 11 | WHAT WAS THE WEIGHT OF THIS CHILD AT BIRTH? (in Kg)  Omwana yali azitowa kilo meka wewamuzalila? | …………………  < 2.5Kg  ≥ 2.5Kg  Unknown | 1  2  3 |  |
| 12 | HOW OLD WAS THIS PREGNANCY WHEN YOU DELIVERED? (37 weeks is approximately 9 months)  Olubutto lwonno omwana lwali lutuse ku mwezi emeka we wamuzalila? | Before 37 weeks  At or after 37 weeks | 1  2 |  |
| 13 | WHAT TYPE OF CLEFT DOES THE CHID HAVE? (Request to check the child’s hospital card)  Kankebere ko ku kadi ye ekyika kyo’bulemu omwana ono bwalina. | Unilateral CL  Bilateral CL  Incomplete Unilateral CLP  Complete Unilateral CLP  Complete Bilateral CLP | 1  2  3  4  5 |  |
| 14 | WAS THIS CHILD BORN WITH ANY OTHER DISABILITY? (Request to check the child’s hospital card)  Kankebere ko ku kadi ye oba omwana ono yazalibwa no’bulemu obulala. | Pierre Robin sequence  Other (Specify) ……………….  N/A | 1  2  9 |  |
| 15 | WHAT IS YOUR HIV STATUS?  Olina akawuka kamukenenya? | Positive  Negative  Don’t Know  N/A | 1  2  3  9 |  |
| 16 | IF POSITIVE, DOES YOUR CONDITION INTERFERE WITH THE CHILD’S FEEDING?  Endwadde eno ekutataaganya mu ndiisa y’omwana? | Yes  No  N/A | 1  2  9 |  |
| 17 | IF YES, HOW DOES IT INTERFERE WITH THE CHILD’S FEEDING?  Nyonyola engeri endwadde eno gy’ekututataganya mu ndiisa y’omwana? | Fail to breastfeed child  Fail to access food  Separates you from child  Others (Specify) ……………………………..  N/A | 1  2  3  4  9 |  |
| 18 | HOW DO YOU KNOW THAT A CHILD IS MALNOURISHED?  Omanya otya nti omwana azingamye? | Failure to grow in length/height  Failure to gain weight  Failure to feed  Swollen feet  Pot belly  Others (Specify)  ……………………………...  N/A | 1  2  3  4  5  6  9 |  |
| 19 | WHAT ARE THE RECOMMENDED FEEDING PRACTICES FOR CHILDREN FROM BIRTH TO 2 YEARS?  Omwana wandimulisiiza otya okuva nga w’akamuzala paka myaka ebbiri? | Initiate breastfeeding early  Exclusively breastfeed for 6 months  Introduce soft foods at 6 months  Continue breastfeeding until 2 years or beyond  Others (Specify)  ………………………………  N/A | 1  2  3  4  5  9 |  |
| 20 | HAVE YOU EVER ATTENDED A FEEDING COUNSELLING SESSION?  Wali Weetabyeko mu kosomesabwa ku ndiisa y’abaana | Yes  No | 1  2 |  |
| 21 | WHEN WAS THIS?  Kino kyali wo ddi? | During Antenatal Care  During Postnatal care  When child was sick  Other situation (Specify)………………..  ……………………………..  N/A | 1  2  3  4  9 |  |
| 22 | WHERE WAS THIS?  Kyali ludda wa? | Health Facility  Community gathering  Others (Specify)…….  ………………………………  N/A | 1  2  3  9 |  |
| 23 | HAVE YOU RECEIVED ANY TYPE OF SUPPORT FROM YOUR PARTNER/FAMILY MEMBER? | Yes  No | 1  2 |  |
| 2a) | WHAT TYPE OF SUPPORT DID YOU RECEIVE? | Financial  Emotional/counselling  Feeding guidance  Health related  Others (Specify)…….  ………………………………  N/A | 1  2  3  4  5  9 |  |

**Section 2: Infant and Young Child Feeding Questions**

| **No.** | **QUESTIONS** | **RESPONSES** | **Skip pattern** | |  | **CODE** |
| --- | --- | --- | --- | --- | --- | --- |
| 23 | DID YOU EVER BREAST FEED OR BREASTMILK FEED THIS CHILD? (Breastfeeding includes breastmilk feeding)  Ono omwana wali omuyonseza ko? | Yes | Go to 25 | | 1 |  |
|  |  | No | Go to 24 | | 2 |  |
| 24 | IF NO, WHY NOT?  Bwoba tomuyonsanga, nsonga ki ekugaana okumuyonsa? | Child could not latch/position on breast  Milk escapes through nose/  Child chokes  Mother had no breastmilk  HIV Exposed/infected  Culturally unacceptable  Mother doesn’t know how to breastfeed child with cleft  Others (Specify)………………………….  N/A | | | 1  2  3  4  5  6  7  8  9 |  |
| 25 | IF YES, WAS THIS CHILD BREASTFED OR BREASTMILK FED YESTERDAY DURING THE DAY OR AT NIGHT?  Omwana yayonseko eggulo, mulunaku oba ekyiro? | Yes  No  N/A | | | 1  2  9 |  |
| 26 | IF YES, HOW LONG AFTER BIRTH DID YOU PUT THIS CHILD TO THE BREAST?  Ng’omazze okumuzalaa, wamala banga lyenkana wa omwana okumuteka ku bbere? | Immediately  Within the first 1 hour  Within the first 24 hours  Within days  N/A | | | 1  2  3  4  9 |  |
| 27 | DID THIS CHILD HAVE ANY OF THE FOLLLOWING LIQUIDS DURING THE DAY OR AT NIGHT?  (For every liquid category, Write 1 if it was taken, 2 if it wasn’t taken and 9 if she doesn’t know)  (For the milk products B, C and E write the number of times it was taken)  Omwana yanwedde ku byokunwya bino wamanga mu lunaku oba ekyiro kya jyo? | LIQUID CATEGORY | | Yes=1  No=2  N/A= 9 | |  |
|  |  | **A.** Plain water  **B.** Infant formula like NAN  **C.** Fresh animal milk, powdered milk  **D.** Juice or juice drinks  **E.** Yoghurt/ Bongo  **F.** Thin porridge  **G.** Any other liquids | | ………..  ………..  ………..  ………..  …………  …………  ………… | |  |
| 28 | HOW MANY TIMES YESTERDAY DURING THE DAY OR NIGHT DID THE CHILD CONSUME ANY OF THE FOODS **B**, **C** AND **E** ABOVE?  Omwana yanwedde ku byokunwya bino wamanga emmirundi emyeka? Olunaku olwajyo mu lunaku oba ekyiro? | **B.** Infant formula like NAN  **C.** Fresh animal milk, powdered milk  **E.** Yoghurt/ Bongo | | …………  …………  ………… | |  |
| 29 | PLEASE DESCRIBE EVERYTHING THAT THIS CHILD ATE YESTERDAY SINCE WAKING UP, DURING THE DAY OR NIGHT? (Probe for ingredients in mixed dishes.) (As she recalls, write 1, 2 or N/A if the food group was eaten, not eaten or doesn’t know respectively)  (Score 1 point if you wrote 1 for yes.)  Mbulilako ebyokulya byona omwano ono byeyalidde okuva bweyazukusse gyo, emisana n’ekyiro. | FOOD GROUPS | | Yes=1  No= 2  N/A= 9 | Score |  |
|  |  | **A.** Grain foods (Porridge, rice), Roots and tubers (Potatoes, cassava)  **B.** Beans, peas, nuts  **C.** Dairy (milk, yoghurt, ghee)  **D.** Meats, fish, chicken, liver, organ meats, fish & dried fish  **E.** Eggs  **F.** Ripe Mangoes, Pawpaw, pumpkin, carrots, (Dark green vegetables, Nakati, Ddodo) Vit A fortified oil  **G.** Other fruits and vegetables | | ……..  …..………..  …..…  …………  … |  |  |
| 30 | DID THIS CHILD EAT ANY SOLID, SEMI-SOLID, OR SOFT FOODS YESTERDAY DURING THE DAY OR NIGHT?  IF YES, WHAT KIND OF SOLID, SEMI-SOLID OR SOFT FOODS? (Go back above and record any new food groups)  Omwana wamugabiridde ko ku mmere egonda olunaku olwa jyo n’ekyiro? Mmere kyi? | Yes  No  N/A | | | 1  2  9 |  |
| 31 | HOW MANY TIMES DID THIS CHILD EAT SOLID, SEMI-SOLID OR SOFT FOODS YESTERAY DURING THE DAY OR AT NIGHT?  Omwana wamugabiridde ko ku mmere egonda emirundi emeka olunaku olwa jyo n’ekyiro? | Number of times  N/A | | | …  9 |  |

**Section 3:** **Consists of questions about the feeding techniques used by mothers in feeding children with cleft lip and or palate**

| **No.** | **QUESTIONS** | **RESPONSES** |  | **CODE** |
| --- | --- | --- | --- | --- |
| 32 | DID YOU USE ANY UTENSILS TO FEED THIS CHILD BREASTMILK, OTHER LIQUIDS AND FOODS YESTERDAY?  Okozesezako ebyokuliisa ng’owa omwana ammabere, ebyokunywa oba emmere olunaku olwa jyo? | Yes  No  N/A | 1  2 |  |
| 33 | IF YES, WHAT UTENSIL DID YOU USE TO FEED THIS CHILD YESTERDAY?  Wakozeseza Kyakuliisa kyi kubino ebiri wamanga? | Spoon  Cup  Bottle with a nipple  Specialized bottle (Soft bottle, Haberman feeder)  Specialized cup (nifty cup)  Nasal Gastric tube  N/A | 1  2  3  4  5  6  9 |  |
| 34 | WHY DID YOU USE THE UTENSIL YOU MENTIONED ABOVE?  Lwakyi walonze okukozesa ebyokuliisa ebyo? | Less spillage  Improved feeding time  Ease of feeding  No escape through the nose  Others…………………………  N/A | 1  2  3  4  5  9 |  |

**Thank you for your patience and cooperation**
