# Supplementary material for: “Mine did not breastfeed”, mothers’ experiences in breastfeeding children aged 0 to 24 months with oral clefts in Uganda
Source: BMC Pregnancy Childbirth. 2021 Jan 30;21:100. doi: 10.1186/s12884-021-03581-3 (PMC7847043; doi:10.1186/s12884-021-03581-3)
Supplement: Supplementary file 2 — Additional file 2. In Depth Interview guide. In Depth Interview guide for interviewing mothers (or fathers/family members) on their experiences and challenges in feeding their children with oral clefts. [file 12884_2021_3581_MOESM2_ESM.docx]

**In Depth Interview (IDI) guide**

IDIs with mothers (or fathers/family members) on their experiences and challenges in feeding their children with oral clefts.

You’re welcome, thanks for agreeing to be interviewed for this study. My name is …………… I work with MakSPH. Can you please introduce yourself? The reason I am talking to you today is to understand your experiences and challenges as a mother (or father/family member) in feeding your child with cleft

*Nkwaniriza nyabo oba ssebo. Webale kukiriza kwetaba mu kunonyereza kuno. Amanya nze………. Nkola ne MakSPH. Nsaaba weyanjule. Twagala kunonyereza kumbera n’ebizibu bamaama byebayitamu mu kuliisa abaana obalina obulemu.*

| District: |  | Region: |  |
| --- | --- | --- | --- |
| Type of IDI: |  | Name of Interviewer: |  |
| Name of respondent: |  | Date: |  |
| Age of respondent: |  | Venue: |  |
| Marital status: |  | Start time: |  |
| Child’s type of cleft: |  | End time: |  |

**Section 1: Ice breaker**

1. Tell us about your family and your children in general

*Tubulileko ko ku baana bo ne famile yo*

2. Briefly tell us one thing your child does that makes you smile

*Tubulile yo akantu kamu omwana wo kakola akusanyusa*

**Section 2: Key Questions**

3. How would you describe your journey as a mother (or father/family member) to a child with cleft?

*Tubulileko ku lugendo lwo ngo’muzadde w’omwana alina obulemu buno*

4. Describe your experiences in feeding your child with cleft lip and or palate since the day you became a mother (or the child was born)

Nsaba otunyumize embera gy’oyitamu mu kuliisa omwana ono alina obulemu buno okuva olunaku lweyazaalibwa

Probe: a) how have you been feeding the child since he/she was born?

5. Describe the challenges you experienced in feeding your child since the day he/she were born

*Nsaba otunyonyole ebizibu by’oyitamu mu kuliisa omwana ono alina obulemu buno okuva olunaku lwewamuzaala*

Probe: a) what challenges did you as the mother experience? *(Biziibu kyi byosanze gwe nga maama oba taata?)*

b) What challenges did your child experience? *(Biziibu kyi omwana byasanze?)*

6. How did these challenges make you feel?

*Ebizibu bino byakuyisa bitya?*

7. How did you cope with these challenges?

Ebizibu bino wabiyitamu otya; wakol’otya?

**Section 3: Additional comments**

8. In your view, what would you do different in terms of feeding your child if you were given another chance?

*Ssinga obadde osoboola okudaayo emabegga, wandi kyussizamu kyi mu kuliisa kw’omwana wo ono?*

9. Given the experiences you’ve just described to me, what advice would you give other mothers (or fathers) in feeding their children with cleft?

*Okusinzira ku mbera jy’ombulidde, magezi kyi ge wandi wadde ba maama (oba ba taata) abalala mu nsonga z’endya y’abaana abalina obulemu buno?*

**Section 4: Summary statement**

10. If we could just go over the discussion we have just had… (summarize the discussion and ask if that is how the discussion flowed)

*Katubiddemu katono byetwogeddemu*

8. Is there anything else you would like discuss with us on feeding children with cleft?

*Waliyo ensonga endala ze wandiyagadde okwogelako nange ku by’endiisa y’abaana bano?*

**Thank you for your cooperation (Tweyanziza nyo)**
