# Supplementary material for: “Mine did not breastfeed”, mothers’ experiences in breastfeeding children aged 0 to 24 months with oral clefts in Uganda
Source: BMC Pregnancy Childbirth. 2021 Jan 30;21:100. doi: 10.1186/s12884-021-03581-3 (PMC7847043; doi:10.1186/s12884-021-03581-3)
Supplement: Supplementary file 3 — Additional file 3. Focus Group Discussion guide. Focus Group Discussion guide for discussing with mothers to children with oral clefts on feeding assistance, healthcare services and social support received. [file 12884_2021_3581_MOESM3_ESM.docx]

**Focus Group Discussion guide**

FGDs with mothers to children with oral clefts on feeding assistance, healthcare services and social support received

You’re welcome, thanks for agreeing to be in this discussion for this study. My name is …………… I work with MakSPH. Can you please introduce yourselves?

*Mwebale kugya, n’okutwegatako mu lukungaano luno. Amanya nze……. Nva MaKSPH. Nsaba mweyanjule.*

The purpose of this discussion with you today is to understand your opinion on the feeding assistance, healthcare services and social support received for your children with cleft.

*Ekyigendelelwa ky’olukungaana luno ya kumanya buyambi bwemufuna mu by’endya, ebyobulamu n’obuyambi obulaala eri abaana bamwe abalina obulemu.*

**Ground rules *(Amateeka)*:**

- We would like you to do the talking *(Tusaba mwetabe mukwogela)*
- There are no right or wrong answers *(Tewali nsonga ntuufu oba nkyamu)*
- What is said here stays here *(Ebyogeredwa wano bisiggala wano)*
- We will audio record the discussion *(Amaloboozzi tugenda gakwata ku recorder eno)*

**Preliminary information**

| Type of FGD: |  | Moderator: |  |
| --- | --- | --- | --- |
| FGD date: |  | Note taker: |  |
| No. of participants: |  | Language used: |  |
| Start time: |  | Venue: |  |
| End time: |  |  |  |

**Section 1: Ice breaker**

1. Let us discuss the one thing your children do that makes you smile

*Mutubulile yo akantu kamu abaana bamwe kebakola akabausanyusa*

**Section 2: Key Questions**

4. Can someone describe her life since she become a mother to a child with cleft

*Tunyumiz’eko kubulamu bwo okuva bwe wazaala omwana wo ngaliko obulemu*

3. Can someone describe her experience in getting assistance for feeding her child with cleft

*Tubulileko ku mbera zoyissemu mu kunonya obuyambi mu kuliisa omwana wo ono*

Probes: a) Please describe in detail any help/guidance you’ve ever got *(Ofunye yo ko ku buyambi obwekyika kyona?)*

b) Please describe where you found this help and the kind of people who gave it *(Tubuulile gy’ewabugya era abantu ababukuwa)*

c) How did this help contribute to your feeding experience with your child? *(Obuyambi buno bwakuyamba butya mu ndiisa y’omwana wo ono?)*

5. Describe your experience in getting assistance for health problems for your child with cleft

*Tubulile ko ku mbera gyoyiisemu mu kunoonya obuyambi mu bizibu eby’obulamu bw’omwana wo ono*

Probes: a) Please describe in detail any health services you’ve ever got; whether it was diagnosis, surgery, nutrition services or dentist services. (*Tunyonyole obujanjabi bwona bwewali ofunye ko ku eby’obulamu bw’omwano wo ono)*

b) Please describe where you found these health services *(Tunyonyole obujajabi buno gy’ewabusanga)*

c) Describe the kind of people who offered them *(Ng’abani ababukuwa?)*

d) How did this help contribute to the health of your child? *(Bwayamba butya obulamu bw’omwana ono?)*

6. Describe your experience in getting assistance for your child with cleft from family or community members

*Tubulile ko ku mbera gyoyiisemu mu kunoonya obuyambi obulala bw’omwana wo ono okuva mu maka gy’ova, ab’ekyika, oba ab’okukyaalo kyo*

Probes: a) Describe how your family/community responded to you and your child *(Abantu bo abo bakitwala batya ng’ozadde omwana ono, era baabayissa batya mwembi?)*

b) Please describe any help/guidance you’ve ever got *(Tunyonyole obuyambi bwona bwewali ofunye ku mwana ono okuva eri abantu bo)*

c) Please describe where you found this help (Wabujya wa obuyambi buno?)

d) Describe the kind of people who gave it (Ng’abaani ababukuwa?)

e) In your opinion, how did this help contribute to you looking after your child? *(Olowozza obuyambi buno bwakuyamba butya mu ndabirira y’omwana ono?)*

**Section 3: Additional comments**

7. Given the experiences you’ve just described to me, what kind of services should be provided for children with cleft?

*Okusinzira kubyongambye, olowooza embera z’abaana abalina obulemu tusobola zilongosa mu tutya?*

**Section 4: Summary statement**

8. If we could just go over the discussion we have just had… (summarize the discussion and ask if that is what the respondent meant)

*Katubiddemu katono byetwogeddemu*

9. Is there anything else you would like discuss with us in terms of support for children with cleft?

*Waliyo ensonga endala ze wandiyagadde okwogelamu ko nange nga zikwata ku buyambi bw’abaana bano?*

**Thank you for your cooperation**

***Tweyanziza nyo***
